# Combined Expression of HGFR with Her2/neu, EGFR, IGF1R, Mucin-1 and Integrin α2β1 Is Associated with Aggressive Epithelial Ovarian Cancer

**DOI:** 10.3390/biomedicines10112694

**Published:** 2022-10-25

**Authors:** Bastian Czogalla, Katharina Dötzer, Nicole Sigrüner, Franz Edler von Koch, Christine E. Brambs, Sabine Anthuber, Sergio Frangini, Alexander Burges, Jens Werner, Sven Mahner, Barbara Mayer

**Affiliations:** 1 Department of Obstetrics and Gynecology, University Hospital, Ludwig-Maximilians-University Munich, Marchioninistraße 15, 81377 Munich, Germany; 2 German Cancer Consortium (DKTK), Partner Site Munich, Pettenkoferstraße 8a, 80336 Munich, Germany; 3 Department of General, Visceral and Transplant Surgery, University Hospital, Ludwig-Maximilians-University Munich, Marchioninistraße 15, 81377 Munich, Germany; 4 Gynecology and Obstetrics Clinic, Klinikum Dritter Orden, Menzinger Straße 44, 80638 Munich, Germany; 5 Department of Obstetrics and Gynecology, Klinikum Rechts der Isar, Technical University Munich, Ismaninger Straße 22, 81675 Munich, Germany; 6 Department of Obstetrics and Gynecology, Starnberg Hospital, Oßwaldstraße 1, 82319 Starnberg, Germany; 7 Department of Obstetrics and Gynecology, Munich Clinic Harlaching, Sanatoriumsplatz 2, 81545 Munich, Germany

**Keywords:** epithelial ovarian cancer, prognosis, immunohistochemistry, HGFR, Her2/neu, EGFR, IGF1R, Muc-1, α2β1

## Abstract

Hepatocyte growth factor receptor (HGFR), also known as c-mesenchymal–epithelial transition factor (c-MET), plays a crucial role in the carcinogenesis of epithelial ovarian cancer (EOC). In contrast, the mechanisms contributing to aberrant expression of HGFR in EOC are not fully understood. In the present study, the expression of HGFR with its prognostic and predictive role was evaluated immunohistochemically in a cohort of 42 primary ovarian cancer patients. Furthermore, we analyzed the dual expression of HGFR and other druggable biomarkers. In the multivariate Cox regression analysis, high HGFR expression was identified as an independent prognostic factor for a shorter progression-free survival (PFS) (hazard ratio (HR) 2.99, 95% confidence interval (CI95%) 1.01–8.91, *p* = 0.049) and overall survival (OS) (HR 5.77, CI95% 1.56–21.34, *p* = 0.009). In addition, the combined expression of HGFR, human epidermal growth factor receptor 2 (Her2/neu), epithelial growth factor receptor (EGFR), insulin-like growth factor 1 (IGF1R), Mucin-1 and Integrin α2β1 further significantly impaired PFS, platinum-free interval (PFI) and OS. Protein co-expression analyses were confirmed by transcriptomic data in a large, independent cohort of patients. In conclusion, new biomarker-directed treatment targets were identified to fight poor prognosis of primary EOC.

## 1. Introduction

Epithelial ovarian cancer (EOC) is one of the most lethal tumor entities [1]. Lack of adequate screening methods and rising resistances towards chemotherapy over the clinical course contribute to a low 5-year survival rate at around 45% [1,2]. The standard of care for advanced EOC is a radical cytoreductive surgery followed by adjuvant platinum-based chemotherapy and maintenance targeted therapy such as anti-angiogenic antibody, bevacizumab or poly-ADP-ribose-polymerase inhibitors [3]. Even though initial response rates are between 60–80%, the majority of patients will develop therapy resistance, leading to subsequent recurrence or progression of disease. Therefore, translational research approaches must elucidate molecular mechanisms in the carcinogenesis of EOC for developing new prognostic and therapeutic strategies. Taking the heterogeneity of EOC into account appears crucial for future personalized cancer therapy.

Hepatocyte growth factor receptor (HGFR), also known as c-mesenchymal–epithelial transition factor (c-MET), is a tyrosine kinase receptor. It regulates important cellular processes, such as differentiation, proliferation, cell cycle, motility and apoptosis, through its sole ligand hepatocyte growth factor (HGF) [4,5]. Despite its physiological functions, prolonged or continuous HGFR signaling activity with subsequent catalytic activation of signal transduction cascades is involved in the carcinogenesis of liver, lung, breast, pancreatic, gastric, head and neck, renal and cervical cancer [6,7,8,9,10]. With growing evidence of important crosstalks between HGFR and other cell surface receptors and proteins attributing to carcinogenesis, the characterization of this molecular mechanism is pivotal to explore new therapeutical approaches [11].

Former studies could demonstrate enhanced HGFR expression in EOC correlating with higher histological grading, distant metastasis and impaired survival rates [12,13,14,15,16,17,18]. HGFR levels in the blood are an independent prognostic biomarker for ovarian cancer [19]. Furthermore, high HGFR expression is associated with *TP53* mutations, pathognomonic for high-grade EOC [20]. Nevertheless, the mechanisms contributing to increased expression of HGFR in EOC are not fully understood so far. 

Molecular targeting of HGFR and associated cell surface receptors and proteins could be a promising strategy for new therapeutical approaches in EOC, which remains to be explored. In the present study, the expression of HGFR in EOC with its prognostic and predictive role was evaluated. Furthermore, we analyzed the effect of the combined expression of HGFR and the growth factor receptors estrogen receptor alpha (ERα), progesterone receptor (PR), human epidermal growth factor receptor 2 (HER2/neu), epithelial growth factor receptor (EGFR) and insulin-like growth factor 1 (IGF1R) as well as the cell adhesion molecules Mucin-1, CD44v6 and Integrin α2β1 on EOC patients’ survival.

## 2. Materials and Methods

### 2.1. Study Population

Forty-two patients with a primary, chemonaive ovarian, fallopian tube or peritoneal cancer from the SpheroID-Study were included. Patients with another neoplasia within the last five years were excluded. Informed consent was obtained from all patients in the study. The study was approved by the Ethics Committee of Ludwig Maximilians University, Munich, Germany (approval number 278-04). Between September 2012 and January 2015, the patients were recruited in five clinics: University Hospital, LMU Munich (n = 16); Klinikum Dritter Orden (n = 13); Klinikum rechts der Isar; Technical University Munich (n = 7); Munich Clinic Harlaching (n = 4); and Starnberg Hospital (n = 2). Standardized surgery and pathological analysis were performed by the respective clinics. Relevant clinicopathological data for statistical analyses were selected from routine reports and delivered in a pseudonymized form. Survival analysis was performed after chemotherapy. All patients received 6 cycles of a carboplatin–paclitaxel treatment. Progression-free survival (PFS) was defined as the time from surgery to progression or relapse. Platinum-free interval (PFI) was defined as the time from end of the platinum-based chemotherapy to progression or relapse. Overall survival (OS) was defined as the time from surgery to death. Data from patients without death and progression/relapse were censored at the date of their last visit.

### 2.2. Immunohistochemistry

After surgical resection, tumor samples were snap-frozen in liquid nitrogen. Serial cryosections (5 µm) were performed. The samples were stained immunohistochemically with the avidin–biotin–peroxidase method as described in detail in [21,22,23]. Briefly, after fixation and blocking of unspecific Fc receptors and endogenous biotin, tissue sections were stained with the primary antibodies for one hour. Secondary biotinylated antibodies and peroxidase-conjugated streptavidin (Dianova, Hamburg, Germany) were incubated for 30 min. The antigen–antibody reaction was visualized by incubation in 3-amino-9-ethylcarbazole pH 4.7 (Sigma-Aldrich, Steinheim, Germany) peroxidase solution for eight minutes. Tissue sections were counterstained in Mayer’s hematoxylin (Merck, Darmstadt, Germany) and embedded with Aquatex^®^ (Merck, Darmstadt, Germany). Details about the used antibodies and working concentrations including positive and negative controls are given in Table 1.

### 2.3. Evaluation of Biomarker Expression

Sections were evaluated semiquantitatively. The percentage of positively stained cancer cells was calculated for each analyzed antigen [21,22,23]. Her2/neu expression was evaluated according to breast cancer and gastric cancer guidelines [24,25]. Due to missing standardized cutoffs for other biomarkers, cutoffs were determined according to biphasic distribution or group size (Table 1).

### 2.4. Statistical Analysis

HGFR expression was correlated with clinicopathological factors and other biomarker expressions using the Fisher’s exact two-tailed test. Univariate analysis was evaluated by calculating cumulative survival probabilities with the Kaplan–Meier method and comparing them with the log-rank test. Significant variables identified in the univariate analysis were further considered in the multivariate Cox regression model of survival. Calculations were performed for PFS, PFI and OS. Confirmatory validation studies were performed with the open-access Kaplan–Meier plotter database and the evaluation tools that are available online [26]. *p*-values < 0.05 were defined to be statistically significant. IBM SPSS Statistics 26 (Armonk, NY, USA) was used for all statistical analyses.

## 3. Results

### 3.1. Patient Characteristic

The clinicopathological characteristics of the analyzed ovarian cancer cohort are listed in Table 2. 

Forty-two patients were included in this study. The median age at diagnosis was 66 years (range: 24–83 years). High-grade ovarian cancer in an advanced FIGO (Fédération Internationale de Gynécologie et d’Obstétrique) stage was most frequent. Of all patients, 71% had a complete surgical resection of all macroscopic visible tumor; 93% of patients received carboplatin–paclitaxel-based chemotherapy according to guideline recommendations; and 33% suffered from a relapse after chemotherapy within 12 months. Median OS (overall survival) was 42 months, median PFS (progression-free survival) was 22 months and median PFI (platinum-free interval) was 17 months. 

### 3.2. Prognostic Impact of HGFR Protein Expression

Univariate analysis revealed that high (≥50%) HGFR expression was associated with an impaired PFS (*p* = 0.041), PFI (*p* = 0.048) and OS (*p* = 0.012) (Table 3). Multivariate analysis confirmed high HGFR expression as an independent factor for poor prognosis (PFS: HR 2.99, CI95% 1.01–8.91, *p* = 0.049; OS: HR 5.77, CI95% 1.56–21.34, *p* = 0.009) (Table 3). In the same cohort, the presence of macroscopic residual tumor after surgery was found as an independent factor for short PFS (HR 2.19, CI95% 1.03–4.68, *p* = 0.043) and short OS (HR 8.42, CI95% 1.59–44.61, *p* = 0.012) (Table 3). No significant correlation between HGFR expression and clinicopathological characteristics could be detected (data not shown).

### 3.3. Correlation of HGFR Expression and Other Protein Biomarkers and Prognostic Impact of Combined Expression Profiles

To reveal clinically important correlations of HGFR with other biomarkers, we analyzed the combinations of HGFR with the growth factor receptors ERα, PR, HER2/neu, EGFR and IGF1R as well as the cell adhesion molecules Mucin-1, CD44v6 and Integrin α2β1. We could not find a correlation between the expression of these biomarkers and HGFR in our cohort (Table 4). 

We analyzed the effect of the combined expression of HGFR and the other biomarkers on patients’ survival and detected significant associations with impaired PFS, PFI and OS (Table 5). 

Patients with high expression of HGFR and Her-2/neu showed a shorter PFS (*p* = 0.009) and PFI (*p* = 0.008) compared to the remaining combinations. High expression of HGFR and EGFR was associated with an impaired PFS (*p* ≤ 0.001), PFI (*p* ≤ 0.001) and OS (*p* = 0.011). Likewise, the combination of high HGFR and IGF1R expression correlated with shorter OS (*p* = 0.03). In addition, combined HGFR and MUC-1 expression was associated with impaired PFS (*p* = 0.002), PFI (*p* = 0.003) and OS (*p* < 0.001). Furthermore, patients with high expression of HGFR and Integrin α2β1 showed a shorter PFS (*p* = 0.004) and PFI (*p* = 0.004).

### 3.4. High Co-Expression of MET and the Other Biomarker Genes Is Significantly Associated with Impaired Patient Survival in a Large Independent EOC Cohort

Aiming to validate the prognostic impact of *MET* (HGFR gene) and the other biomarker genes (*ESR1*—ERα gene, *PGR*—PR gene, *ERBB2*—Her-2/neu gene, *EGFR*—EGFR gene, *IGF1R*—IGF1R gene, *MUC1*—MUC-1 gene, *CD44*—CD44 gene, *ITGA2*—Integrin α2 gene) on patients’ survival regarding a larger total of EOC patients, the Kaplan–Meier plotter database was used [26]. For all genes, patients were divided into high- and low-expression groups based on gene-specific cutoff values, before performing analyses concerning OS and PFS (Table 6). 

The analysis revealed that high *MET* expression was associated with an impaired PFS (*p* = 0.018) and OS (*p* = 0.033). Patients with high expression of *MET* and *ERBB2* showed a shorter PFS (*p* = 0.036) and OS (*p* = 0.043) compared to the remaining combinations. High expression of *MET* and *EGFR* was associated with an impaired PFS (*p* = 0.0047). Likewise, the combination of high *MET* and *IGF1R* expression correlated with shorter OS (*p* = 0.048). In addition, combined *MET* and *MUC1* expression was associated with impaired PFS (*p* = 0.018) and OS (*p* = 0.043). Furthermore, patients with high expression of *MET* and *ITGA2* showed a shorter PFS (*p* = 0.003). Corresponding Kaplan–Meier plots are shown in Figure S1.

## 4. Discussion

In the present study, we analyzed HGFR in our EOC cohort and confirmed it as a prognostic and predictive protein biomarker. High HGFR expression was associated with an impaired PFS, PFI and OS and could be proven as an independent prognostic factor for PFS and OS (Table 3). In addition, we analyzed the effect of the combined expression of HGFR and the growth factor receptors ERα, PR, HER2/neu, EGFR and IGF1R as well as the cell adhesion molecules Mucin-1, CD44v6 and Integrin α2β1 on EOC patients’ survival and found significant associations with shorter PFS, PFI and OS (Table 5). The combined high expression of HGFR and other biomarkers is associated with impaired PFS, PFI and OS compared to a high HGFR expression alone (Table 5). These data could be validated on mRNA expression levels in an independent EOC cohort (Table 6).

The role of HGFR in the carcinogenesis of many tumor types is well established. HGFR overexpression in EOC is associated with higher histological grading, higher FIGO stage, distant metastasis and impaired survival rates [12,16,17,27,28,29]. Thus, our data are in line with previous studies. Despite these observed associations, the particular molecular mechanism is not well understood so far. HGFR has crosstalks with several pathways such as PI3K/Akt, BRAF and RAS-MAPK influencing carcinogenesis [30,31]. Regarding HGFR´s pivotal role in cancer, the inhibition of the HGFR/HGF pathway seems to be an interesting therapeutical approach [32,33,34]. Indeed, several inhibitors of the HGFR/HGF pathway were analyzed in different cancer entities including lung, liver and kidney cancer [35,36,37,38]. Cabozantinib, a tyrosine kinase inhibitor targeting HGFR and vascular endothelial growth factor receptor 2 (VEGFR-2), is used in advanced renal cell carcinoma after a phase-three trial demonstrated a significant PFS and OS benefit compared to mTOR inhibitor everolimus [37]. Tivantinib, a selective HGFR tyrosine kinase inhibitor, showed promising results in advanced hepatocellular carcinoma and non-small-cell lung carcinoma, especially in HGFR-overexpressing subgroups [35,38]. 

HGFR inhibition has also been considered in the treatment of EOC [39,40,41,42,43,44]. In vitro HGFR overexpression improved ovarian cancer cell survival and caused resistance to the chemotherapeutics cisplatin and paclitaxel. siRNA knockdown of HGFR restored chemosensitivity in this cell culture model [39]. Furthermore, it was shown that the HGFR-specific inhibitor MK8033 increases chemosensitivity to carboplatin and paclitaxel in different ovarian cancer cell lines [40]. Other studies underlined the antiproliferative and chemosensitizing effect of HGFR inhibition [42,43]. In a phase-two randomized discontinuation trial of cabozantinib in EOC, the HGFR inhibitor demonstrated clinical activity with acceptable toxicities. Seventy patients, 50% platinum refractory/resistant and 83% with at least two former systemic therapies, were enrolled in the study. Cabozantinib showed an objective response rate of 21%, with a median PFS of 5.5 months [41]. Considering the high percentage of platinum-refractory/resistant tumors and former therapy lines, the monotherapy with cabozantinib should be discussed with patients with limited therapeutic options. In contrast, a phase-two trial with 13 patients with recurrent clear cell ovarian, primary peritoneal or fallopian tube cancer could not confirm significant therapeutic effects by cabozantinib monotherapy [44].

Our analysis of the combined expression of HGFR with HER2/neu, EGFR, IGF1R, Mucin-1 and Integrin α2β1 underlines the potential efficiency of dual combination therapies in EOC. Patients with combined overexpression of these factors showed worse prognosis and platinum resistance. Our analysis demonstrated that patients with a combined high expression of HGFR and HER2/neu, EGFR, IGF1R, Mucin-1 and Integrin α2β1 show a very aggressive tumor biology with an impaired median survival compared to HGFR high-expressing tumors alone. These data show that the progression of primary ovarian cancer is a complex multifactorial process involving molecular crosstalks between different signaling pathways [22]. Multi-target biomarker-driven treatment may be a strategy to overcome platinum resistance and poor prognosis.

Moreover, studies of dual targeting demonstrated promising results in tumor growth inhibition in vitro and in vivo [45,46,47,48]. MicroRNA-mediated HGFR/EGFR repression caused an ovarian cancer cell proliferation arrest and an inhibition of tumor growth in an EOC mouse model [45]. Combined HGFR/EGFR expression was associated with an impaired survival in patients with advanced ovarian cancer [48]. Furthermore, combined inhibition by a dual EGFR/HER-2/neu inhibitor (canertinib) and a HGFR inhibitor (PHA665752) resulted in a decreased ovarian cancer cell proliferation [46,47]. To our knowledge, there are no studies on the dual inhibition of HGFR/IGF1R, HGFR/MUC-1 or HGFR/Integrin α2β1 in EOC yet. Thus, further research is needed to elucidate the potential effect of these combined inhibitions.

In conclusion, our present study demonstrated HGFR in combination with HER2/neu, EGFR, IGF1R, Mucin-1 and Integrin α2β1 as candidates for new biomarker-directed treatment strategies in EOC. Validation studies in independent cohorts are needed to prove our findings.

## Figures and Tables

**Table 1:** Biomarkers and antibodies.

Antigen	Clone	Species	Fixation	Use of Kit	wc (μg/mL)	Supplier	Cutoff for Positivity
Primary antibodies							
HGFR	SP44	r	Acetone	-	2.12	Spring Bioscience, Pleasanton, CA, USA	≥50%
ERα	1D5	m	Formalin	+	2.50	Dako, Santa Clara, CA, USA	≥1%
PR	PgR 636	m	Formalin	+	2.50	Dako, Santa Clara, CA, USA	≥1%
HER-2/neu	4B5	r	Acetone	-	1.50	Ventana, Roche, Basel, CH	≥10% (Intensity 2+/3+)
EGFR	H11	m	Acetone	-	2.94	Dako, Santa Clara, CA, USA	≥50%
IGF1R	23-41	m	Acetone	+	4.00	Invitrogen, Carlsbad, CA, USA	≥80%
MUC-1	Ma552	m	Acetone	-	0.50	Monosan, Uden, NL	≥70%
CD44v6	VFF-18	m	Acetone	-	1.00	Affymetrix eBioscience, Santa Clara, CA, USA	≥10%
Integrin α2β1	BHA2.1	m	Acetone	-	2.50	Millipore, Burlington, MA, USA	≥20%
**Positive controls**							
Epithelial Antigen	Ber-EP4	m	Acetone	-	2.50	Dako, Santa Clara, CA, USA	
**Isotype controls**							
MOPC 21	MOPC 21	m		-	5.00	Sigma-Aldrich, St. Louis, MO, USA	
	MOPC 21	m		+	4.00	Sigma-Aldrich, St. Louis, MO, USA	
	DA1E	r		-	2.12	Cell Signaling, Danvers, MA, USA	
**Biotin-conjugated secondary antibodies**							
	111-065-114	g anti r			7.00	Jackson Immunoresearch, West Grove, PA, USA	
	315-065-048	r anti m			0.75	Jackson Immunoresearch, West Grove, PA, USA	

wc: working concentration; m: mouse; r: rabbit; g: goat. All used antibodies’ isotype was IgG1. HGFR: hepatocyte growth factor receptor, Her-2/neu: human epidermal growth factor receptor 2, EGFR: epidermal growth factor receptor, IGF1R: insulin-like growth factor 1, MUC-1: Mucin-1.

**Table 2:** Patient characteristics.

		n or Value	%
**Age**	Mean/median	61/66 years	
	Range	24–83 years	
**FIGO Stage**	I/II	0	0.0
	III	29	69.0
	IV	13	31.0
**pT**	pT2	4	9.5
	pT3	38	90.5
**pN**	pN0	5	11.9
	pN1	28	66.7
	Nx	9	21.4
**cM**	cM0	29	69.0
	cM1	13	31.0
**Primary Tumor Site**	Ovarian	35	83.3
	Fallopian tube	5	11.9
	Peritoneal	2	4.8
**Histological Subtype**	Serous	38	90.5
	Other	4	9.5
**Grading**	Low grade	1	2.0
	High grade	41	98.0
**Ascites**	Yes	36	85.7
	No	6	14.3
**Macroscopic Residual Tumor after Surgery**	None	30	71.4
	<1 cm	7	16.7
	>1 cm	5	11.9
**Lymphatic Vessel Invasion**	Yes	23	54.7
	No	17	40.5
	Missing	2	4.8
**Vascular Invasion**	Yes	6	14.3
	No	34	80.9
	Missing	2	4.8
**First-Line Treatment**	C	3	7.2
	C + P	14	33.3
	C + P + B	25	59.5
**Relapse after Chemotherapy**	<6 months	2	4.8
	6–12 months	12	28.5
	>12 months	28	66.7

n: number of patients, FIGO: International Federation of Gynecology and Obstetrics, p: pathological, c: clinical, T: extent of primary tumor, N: regional lymph node metastasis, Nx: no evaluation of lymph node status, M: distant metastasis, C: Carboplatin, P: Paclitaxel, B: Bevacizumab.

**Table 3:** Univariate and multivariate survival analysis of clinicopathological factors and HGFR.

Variable		PFS				PFI				OS			
	n	Log-Rank		MV Cox Regression		Log-Rank		MV Cox Regression		Log-Rank		MV Cox Regression	
		MS	*p*	HR (CI 95%)	*p*	MS	*p*	HR (CI 95%)	*p*	MS	*p*	HR (CI 95%)	*p*
**Age ≤ 61 years**	19	22	0.965			17	0.970			nr	0.193		
**Age > 61 years**	23	22				17				42			
**<pT3c**	7	27	0.665			22	0.679			45	0.928		
**pT3c**	35	22				17				42			
**pN0**	5	29	0.163			17	0.145			45	0.929		
**pN1**	28	22				22				42			
**cM0**	29	27	0.081			22	0.068			nr	**0.015**	2.25 (0.62–8.18)	0.217
**cM1**	13	16				11				30			
**G1/G2**	2	14	0.579			8	0.610			30	0.843		
**G3**	40	22				17				42			
**Ascites absent**	6	35	0.147			30	0.139			42	0.408		
**Ascites present**	36	19				15				38			
**MR Tumor absent**	30	27	**0.008**	2.19 (1.03–4.68)	**0.043**	22	**0.010**	2.10 (0.99–4.51)	0.057	45	**0.041**	8.42 (1.59–44.61)	**0.012**
**MR Tumor present**	12	13				9				26			
**HGFR low**	19	35	**0.041**	2.99 (1.01–8.91)	**0.049**	30	**0.048**	2.87 (0.97–8.49)	0.057	nr	**0.012**	5.77 (1.56–21.34)	**0.009**
**HGFR high**	23	18				13				35			

PFS: progression-free survival, PFI: platinum-free interval; OS: overall survival, n: number of patients, MV Cox Regression: multivariate Cox regression, MS: median survival (in months) in Kaplan–Meier estimator, HR: hazard ratio, CI: confidence interval, MR Tumor: macroscopic residual tumor, nr: median survival not reached.

**Table 4:** Correlation between HGFR expression and other protein biomarkers.

				HGFR		
			n	<50%	≥50%	*p* #
**Growth Factor Receptor**	**ERα**		42			0.468
		**<1%**		6	4	
		**≥1%**		13	19	
	**PR**		42			0.750
		**<1%**		13	14	
		**≥1%**		6	9	
	**Her-2/neu**		42			1
		**Negative**		14	17	
		**Positive**		5	6	
	**EGFR**		42			0.707
		**<50%**		14	19	
		**≥50%**		5	4	
	**IGF1R**		42			1
		**<80%**		4	4	
		**≥80%**		15	19	
**Cell Adhesion Molecule**	**MUC-1**		42			0.757
		**<70%**		10	10	
		**≥70%**		9	13	
	**CD44v6**		42			1
		**<10%**		14	16	
		**≥10%**		5	7	
	**Integrin α2β1**		42			0.108
		**<20%**		15	12	
		**≥20%**		4	11	

n: number of patients, # *p*-value calculated by Fisher’s exact two-tailed test.

**Table 5:** Univariate survival analysis of dual expression of HGFR and other protein biomarkers.

		PFS		PFI		OS	
	n	MS	*p* *	MS	*p* *	MS	*p* *
**HGFR low**	19	35	**0.041**	30	**0.048**	nr	**0.012**
**HGRF high**	23	18		13		35	
**HGFR** **high/ERα** **high**	19	19	0.186	14	0.199	38	0.051
**Remaining combinations ^#^**	23	30		25		nr	
**HGFR** **high/PR** **high**	9	19	0.481	14	0.489	35	0.281
**Remaining combinations ^#^**	33	27		22		42	
**HGFR** **high/Her-2/neu** **high**	6	16	**0.009**	11	**0.008**	22	0.42
**Remaining combinations ^#^**	36	27		22		42	
**HGFR** **high/EGFR** **high**	4	12	**<0.001**	8	**<0.001**	23	**0.011**
**Remaining combinations ^#^**	38	24		19		42	
**HGFR** **high/IGF1R** **high**	19	18	0.058	13	0.069	35	**0.03**
**Remaining combinations ^#^**	23	30		25		nr	
**HGFR** **high/MUC-1** **high**	13	16	**0.002**	11	**0.003**	26	**<0.001**
**Remaining combinations ^#^**	29	30		25		nr	
**HGFR** **high/CD44v6** **high**	7	16	0.065	11	0.081	38	0.059
**Remaining combinations ^#^**	35	27		22		45	
**HGFR** **high/Integrin α2β1** **high**	11	15	**0.004**	10	**0.004**	27	0.054
**Remaining combinations ^#^**	31	29		25		45	

n: number of patients, MS: median survival (in months) in Kaplan–Meier estimator, * *p*-value calculated by log-rank test. ^#^ Remaining combinations means tumor samples which were HGFR high/Biomarker X low or HGFR low/Biomarker X high or HGFR low/Biomarker X low. nr: median survival not reached.

**Table 6:** Univariate survival analysis of dual expression of *MET* and other biomarker genes.

		PFS		OS		
	n	MS	*p* *	n	MS	*p* *
***MET* low**	192	19	**0.018**	138	49 45	**0.033**
***MET* high**	94	16		188		
***MET *** ** high/*ESR1*** ** high**	161	22	0.29	378	49 49	0.22
**Remaining combinations ^#^**	406	19		229		
***MET *** **high/*PGR*** **high**	155	22	0.17	313	49 49	0.15
**Remaining combinations ^#^**	412	19		294		
***MET *** **high/*ERBB2*** ** high**	124	16	**0.036**	192	45 49	**0.043**
**Remaining combinations ^#^**	109	19		145		
***MET*** ** high/*EGFR*** ** high**	69	17	**0.0047**	69	49 73	0.083
**Remaining combinations ^#^**	49	27		51		
***MET*** ** high/*IGF1R*** ** high**	172	18	0.076	198	45 49	**0.048**
**Remaining combinations ^#^**	157	19		139		
***MET*** ** high/*MUC1*** ** high**	94	16	**0.018**	186	45 48	**0.043**
**Remaining combinations ^#^**	192	19		140		
***MET*** ** high/*CD44*** ** high**	186	18	0.071	192	45 49	0.057
**Remaining combinations ^#^**	143	19		145		
***MET*** ** high/*ITGA2*** ** high**	70	17	**0.003**	70	49 73	0.058
**Remaining combinations ^#^**	51	27		53		

n: number of patients, MS: median survival (in months) in Kaplan–Meier estimator, * *p*-value calculated by log-rank test. ^#^ Remaining combinations means tumor samples which were MET high/Biomarker X low or MET low/Biomarker X high or MET low/Biomarker X low.

## Data Availability

The data presented in this study are available on request from the corresponding author. The data are not publicly available due to protection of detailed patient-related data.

## Supplementary Materials

The following is available online at https://www.mdpi.com/article/10.3390/biomedicines10112694/s1, Figure S1: Gene co-expression analyses of MET and the other biomarker genes.
