# Supplementary material for: Combined Expression of HGFR with Her2/neu, EGFR, IGF1R, Mucin-1 and Integrin α2β1 Is Associated with Aggressive Epithelial Ovarian Cancer
Source: Biomedicines. 2022 Oct 25;10(11):2694. doi: 10.3390/biomedicines10112694 (PMC9687566; doi:10.3390/biomedicines10112694)
Supplement: Supplementary file 1 [file biomedicines-10-02694-s001.zip › biomedicines-1916775-supplementary.pdf]

Supplementary Figure S1: Gene Co-Expression analyses of *MET* and the other biomarker genes

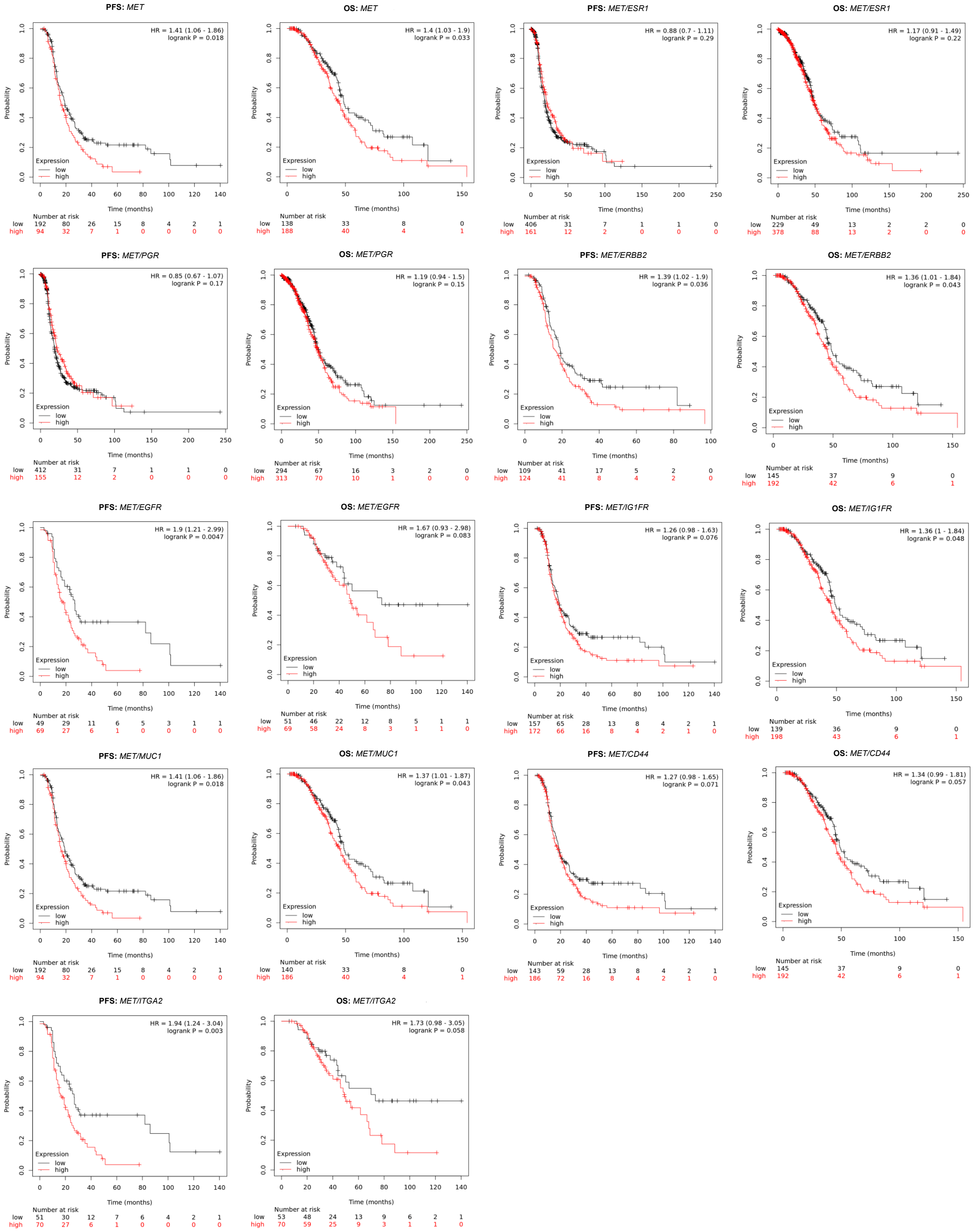

Supplementary Figure S1: Kaplan-Meier plots concerning the gene expression of *MET* (HGFR gene) and the other biomarkers genes (*ESR1* – ERα gene, *PGR* – PR gene, *ERBB2* - Her-2/neu gene, *EGFR* – EGFR gene, *IGF1R* – IGF1R gene, *MUC1* – MUC-1 gene, *CD44* - CD44 gene, *ITGA2* - Integrin α2 gene) on PFS and OS using the Kaplan-Meier plotter database.
